# A within-lake occupancy model for starry stonewort, *Nitellopsis obtusa*, to support early detection and monitoring

**DOI:** 10.1038/s41598-024-52608-0

**Published:** 2024-02-01

**Authors:** Alex W. Bajcz, Wesley J. Glisson, Jeffrey W. Doser, Daniel J. Larkin, John R. Fieberg

**Affiliations:** 1 Minnesota Aquatic Invasive Species Research Center, University of Minnesota, 1992 Folwell Avenue, St Paul, MN 55108 USA; 2 Department of Fisheries, Wildlife, and Conservation Biology, University of Minnesota, 135 Skok Hall, 2003 Upper Buford Circle, St Paul, MN 55108 USA; 3 Department of Integrative Biology, Michigan State University, East Lansing, MI 48824 USA; 4 Ecology, Evolution, and Behavior Program, Michigan State University, East Lansing, MI 48824 USA

**Keywords:** Invasive species, Ecological modelling, Freshwater ecology

## Abstract

To efficiently detect aquatic invasive species early in an invasion when control may still be possible, predictions about which locations are likeliest to be occupied are needed at fine scales but are rarely available. Occupancy modeling could provide such predictions given data of sufficient quality and quantity. We assembled a data set for the macroalga starry stonewort (*Nitellopsis obtusa*) across Minnesota and Wisconsin, USA, where it is a new and high-priority invader. We used these data to construct a multi-season, single-species spatial occupancy model that included biotic, abiotic, and movement-related predictors. Distance to the nearest access was an important occurrence predictor, highlighting the likely role boats play in spreading starry stonewort. Fetch and water depth also predicted occupancy. We estimated an average detection probability of 63% at sites with mean non-*N. obtusa* plant cover, declining to ~ 38% at sites with abundant plant cover, especially that of other Characeae. We recommend that surveyors preferentially search for starry stonewort in areas of shallow depth and high fetch close to boat accesses. We also recommend searching during late summer/early fall when detection is likelier. This study illustrates the utility of fine-scale occupancy modeling for predicting the locations of nascent populations of difficult-to-detect species.

## Introduction

Detecting invasive species early, when management is most tractable^1^, is crucial. However, early detection of small, dispersed populations is difficult^2^—surveyors may not know where to look or even be aware of each new potential threat^3^. Early detection is particularly difficult for aquatic invasive plants because they may not be visible from the surface, may look superficially similar to native species, and can elude capture by common sampling approaches^4,5^. Detection efforts for such species could always benefit from actionable, fine-scale (i.e., within-lake) predictions about where invasives are likeliest to occur, especially early in an invasion^6^. Occupancy models, which relate detection/non-detection data to environmental covariates while accounting for imperfect detection^7^, could yield such predictions^8^. While powerful and well-established for certain taxonomic groups and contexts^9^, occupancy modeling has been applied less often to aquatic invasive species^10^, perhaps because occupancy statuses can change rapidly during an invasion and repeated sampling during a period of closure (an assumption of such models) is uncommon in the context of invasive species monitoring.

**Figure 1:**
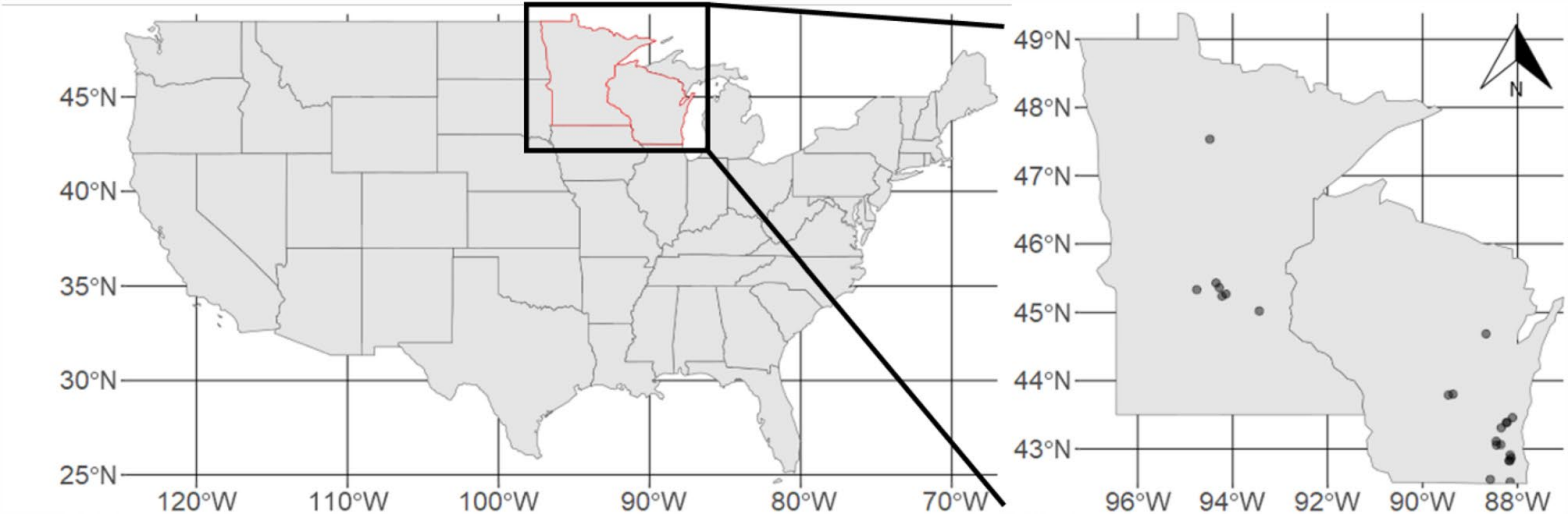
Locations of 23 starry stonewort (*Nitellopsis obtusa*) invaded lakes (gray dots) in Minnesota (left) and Wisconsin (right), USA analyzed in this study.

Here, we leverage the largest data set of within-lake detection/non-detection data compiled to date for starry stonewort (*Nitellopsis obtusa* (Desv. in Loisel.) J. Groves; Characeae), a Eurasian macroalga invasive in North America^11^. Found in nine U.S. states and two Canadian provinces so far, *N. obtusa* has been identified as a significant and growing invasive threat in the Midwest^11^ and thus a high-priority species for early detection and monitoring programs. This species can grow prolifically, potentially displacing native macrophytes, altering habitat for fauna, changing water chemistry, and impairing recreation^2,11–13^ (Fig. 1).

Depth and water turbulence are thought to be key predictors of within-lake occupancy for *N. obtusa*, but how *N. obtusa* occurrence covaries with these two variables is unresolved. For example, prior research has indicated *N. obtusa* may be more common in shallower waters^2,12,14^. However, it remains unclear whether this pattern reflects a true habitat preference, ease of sampling/detection in shallower waters^12^, preferential early colonization of shallower waters^15^, or some combination thereof. *Nitellopsis obtusa* occupancy has also been previously shown to associate with reduced turbulence, i.e., lower fetch^13,16^, but only in a few specific contexts, and the potential for an interaction between depth and fetch (because water mixing is a function of both available wind energy and water depth) has not been explored.

Because *N. obtusa* is not in equilibrium in our study region, where it has only been known since 2014, and because propagule transport on boats and other equipment is thought to be its primary means of spread^17^, predictors related to boater movements also hold promise. Proximity to boat accesses, where boaters could introduce propagules, may be key to explaining patterns of *N. obtusa* occupancy, especially early in an invasion. Access *type* may also matter. Many waterbodies have “public” (i.e., local-, state-, and/or federally managed) and/or “private” (i.e., operated by businesses) accesses that may function as *N. obtusa* introduction points. While public agencies implement spread-mitigation tactics, such as watercraft inspections, at some public accesses^18^, such prevention practices at private accesses are unlikely to be routine, uniform, common, or well-documented. So, while many private accesses may receive less traffic than public ones do, their relative risk could still be elevated.

Imperfect detection of *N. obtusa* using standard sampling methods^19^ (see Methods), even at known-infested points and especially at low abundances, is a known problem^16^. If left unaccounted for, imperfect detection can impair accurate coefficient estimation. Causes of non-detections for *N. obtusa* are likely diverse, among them the taxon’s biology and phenology, its novelty in our region, and its superficially similar appearance to related native macroalgae. These issues suggest several predictors, including time since initial infestation, sampling date, and the densities of other macrophytes (especially of close relatives), could account for variation in *N. obtusa* abundance or detection.

While many approaches have been proposed to address imperfect detection in distribution models^20,21^, most approaches rely on repeated observations from at least a subset of sites, some of which yield both detections and non-detections^7^. Typically, the repeated observations are obtained via multiple visits to sites over some time period when the population is assumed to be closed to immigration/emigration, although alternative approaches exist^22–24^. Only then can an occupancy model potentially distinguish “non-detections” from “true absences” using covariates (but see ^21^ and ^25^). Most of our data were collected by the Minnesota and Wisconsin Departments of Natural Resources as part of systematic macrophyte monitoring; many lakes were sampled in multiple years over a seven-year period starting when *N. obtusa* was first observed in our region (2014–2021), giving us the opportunity to model variance in occupancy and detection probabilities explicitly (Table 1).

**Table 1:** Summary data for the lakes used to construct a within-lake occupancy model for starry stonewort (*Nitellopsis obtusa*). Note that several surveys were conducted in the same year at Koronis, Medicine, and Wind lakes.

State	Lake	Size (ha)	First known year infested	Sample points (# known infested)^a^	Times (Years) surveyed
MN	Koronis	1200	2015	531 (340)	9 (’15-’17, ’18-’19 × 2, ’20–’21)
MN	Moose	243	2016	181 (30)	2 (’17–’18)
MN	West Sylvia	366	2016	169 (0)	2 (’17, ’21)
MN	Grand	263	2017	230 (0)	2 (’18, ’21)
MN	Medicine	374	2018	187 (18)	8 (’18–’21 × 2)
MN	Pleasant	242	2018	139 (0)	1 (’19)
MN	Carnelian	72.8	2020	122 (0)	1 (’20)
WI	Little Muskego	190	2014	553 (89)	3 (’14–’15, ’17)
WI	Big Muskego	870	2015	597 (152)	1 (‘15)
WI	Long	42.5	2015	230 (73)	6 (’15, ’17–’21)
WI	Pike	187	2015	379 (215)	6 (’16–’21)
WI	Silver	49.4	2015	327 (42)	7 (’15–’21)
WI	Green	28.3	2016	235 (43)	6 (‘16–’21)
WI	Wind	372	2017	584 (183)	6 (’17, ’18 × 3, ’19–’20)
WI	Geneva	2190	2018	806 (1)	2 (’19, ’20)
WI	Little Cedar	105	2018	447 (23)	4 (’18–’21)
WI	Emery	12.9	2019	134 (110)	2 (’20, ’21)
WI	Lower Nemahbin	96.7	2019	358 (0)	2 (’19–’20)
WI	Okauchee	490	2019	651 (1)	1 (’19)
WI	Pewaukee	986	2019	695 (24)	1 (’20)
WI	Kilby	17.8	2020	125 (2)	1 (’21)
WI	Camp	178	2021	477 (9)	1 (’21)
WI	Pine	87.8	2021	262 (4)	1 (’21)

^a^Repeated surveys typically included the same point locations; however, this was not always the case. Hence, the number of points shown here may exceed the number sampled during any one survey event. Additionally, GPS measurement error sometimes occurred across surveys on the same lake–to account for this error, we treated observations taken ≤ 10 m of each other (≪ the grid spacing on any lake) as being from the same point. Counts of infested points were derived by summing the numbers of points with ≥ 1 detections across all surveys.

We formulated a multi-season, single-species occupancy model to inform early monitoring and detection of *N. obtusa* in our region and to bolster our understanding of its habitat preferences and mechanisms of spread for the benefit of future control and prevention efforts. We hypothesized that within-lake *N. obtusa* occurrence would positively correlate with (1) greater proximity to the closest access (perhaps especially when this access is private), (2) higher local density of accesses (perhaps especially private ones), (3) shallower depths, and (4) lower fetches, in keeping with past study of this species. We also expected *N. obtusa* detection probability to be greater (5) later in the growing season and when (6) other macrophytes, (7) particularly other Characeae, were less abundant.

## Results

As predicted, *time since first infestation* was positively associated with *N. obtusa* occupancy ($\widehat{\beta }$ = 1.69; 95% CI 1.44–1.96; Table 2), indicating a systematic increase in occupancy probability over time as *N. obtusa* spreads within a lake. There was substantial variation in occupancy probability across lakes (random lake-level intercept variance = 15.0; 95% CI 5.69 to 35.4), indicating important lake-level processes could be missing from our model and/or that infestations were first discovered at different time points in the invasion process. Variance of the local spatial random effects was also large (mean σ^2^ = 9.14; 95% CI 7.57 to 9.97), indicating spatial variation in occupancy within lakes that could not be explained by covariates in our model. Together, these results suggest that understanding of both large-scale (lake-level) and fine-scale (within a few hundred meters) spatial processes must be improved to fully predict *N. obtusa* occupancy using predictors alone.

**Table 2:** Model results for single-species, multi-season occupancy models (a base model and three deviations; see Methods) for starry stonewort (*Nitellopsis obtusa*) using data from 23 invaded lakes in Minnesota and Wisconsin, USA. Predictor data were mean-centered and scaled prior to analysis.

Level	Variable	Posterior mean (95% CIs)			
		Base model	Deviation 1	Deviation 2	Deviation 3
Occupancy	**Intercept**	− − 5.85 (− 7.40, − 4.12)	− 4.81 (− 6.37, − 2.98)	− 5.58 (− 7.27, − 3.60)	− 5.98 (− 7.50, − 4.20)
	**Depth**	− 1.60 (− 1.88, − 1.33)	− 1.64 (− 1.94, − 1.37)	− 1.77 (− 2.06, − 1.48)	− 1.60 (− 1.87, − 1.33)
	**Time since first infestation (years)**	1.69 (1.44, 1.96)	1.74 (− 1.48, 2.01)	1.77 (1.49, 2.07)	1.70 (1.44, 1.96)
	**Distance to nearest access**	− 1.12 (− 1.66, − 0.601)	− 1.06 (− 1.60, − 0.540)	− 1.17 (− 1.73, − 0.622)	− 1.50 (− 2.17, − 0.843)
	Nearest access type (private = 1)	0.871 (− 0.0892, 1.82)	0.776 (− 0.158, 1.71)	0.425 (− 0.537, 1.39)	0.891 (− 0.0232, 1.83)
	Access number-Public	− 0.208 (− 0.680, 0.253)	− 0.211 (− 0.681, 0.261)	− 0.0857 (− 0.559, 0.384)	− 0.214 (− 0.687, 0.251)
	Access number-Private	− 0.0333 (− 0.356, 0.291)	− 0.0291 (− 0.362, 0.304)	− 0.154 (− 0.505, 0.193)	− 0.0384 (− 0.360, 0.284)
	Fetch	0.243 (− 0.266, 0.766)	0.214 (− 0.317, 0.724)	0.103 (− 0.428, 0.631)	0.253 (− 0.259, 0.750)
	Depth*fetch	− 0.573 (− 0.864, − 0.290)	− 0.594 (− 0.903, − 0.297)	− 0.648 (− 0.954, − 0.347)	− 0.572 (− 0.869, − 0.287)
	Distance*time	NA	NA	NA	0.681 (0.0723, 1.30)
Detection	**Intercept**	0.534 (0.419, 0.651)	0.527 (0.413, 0.648)	0.570 (0.438, 0.710)	0.536 (0.421, 0.657)
	**Overall plant density**	− 0.348 (− 0.444, − 0.253)	− 0.347 (− 0.443, − 0.253)	− 0.215 (− 0.323, − 0.107)	− 0.350 (− 0.445, − 0.260)
	**Characeae density**	− 0.165 (− 0.239, − 0.0910)	− 0.168 (− 0.243, − 0.0927)	− 0.249 (− 0.329, − 0.168)	− 0.164 (− 0.238, − 0.0888)
	Day of the year	0.0340 (− 0.0368, 0.107)	0.0336 (− 0.0384, 0.106)	− 0.190 (− 0.267, − 0.113)	0.0345 (− 0.0355, 0.107)

Significant predictors (i.e., those for which 0 did not fall within the 95% Credible Interval) in the base model are shown in bold. Deviation 1 excluded data from five lakes with no *N. obtusa* detections across any surveys. Deviation 2 excluded data from Wind Lake (Wisconsin, USA), where the areas around a private and a public access were heavily infested and uninfested, respectively. Deviation 3 added an interaction term between *distance to the nearest access* and *time since infestation* to the occupancy level of the model (see Eq. (2) in the text).

**Figure 2:**
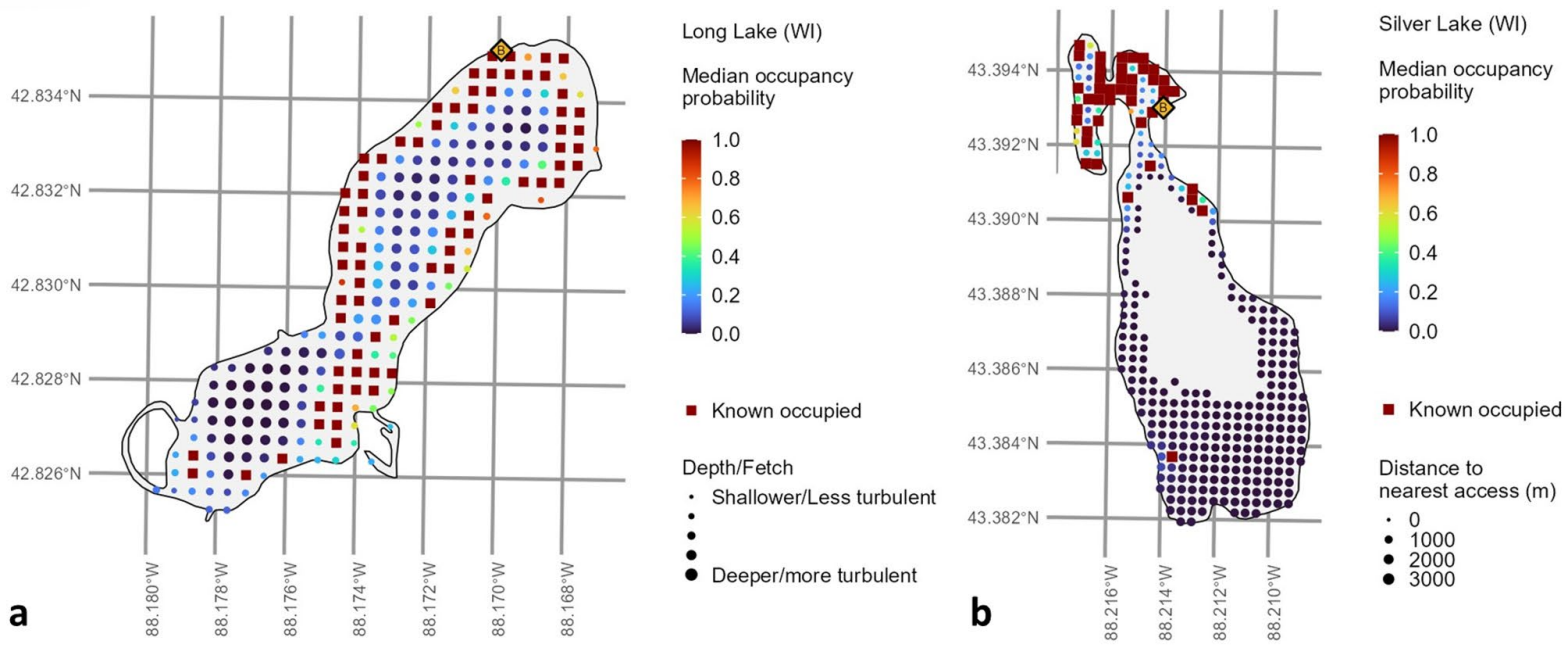
Predicted median occupancy probabilities for starry stonewort (*Nitellopsis obtusa*) in 2021 for (**a**) Long Lake (Wisconsin, USA) and (**b**) Silver Lake (Wisconsin, USA) from a multi-season, single-species occupancy model. Point size reflects variation in significant covariates: (**a**) *fetch by depth interaction* (i.e., greater values indicate deeper and/or more wind-mixed waters) and (**b**) *distance from the nearest access*. Dark red squares indicate sites at which starry stonewort has been detected at least once within our data set. Yellow diamonds indicate public boat accesses.

The interaction between *fetch* and *depth* was significant ($\widehat{\beta }$ = − 0.573; 95% CI − 0.864 to − 0.290; Table 2; Fig. 2a), indicating a complex relationship between these predictors and *N. obtusa* occupancy. Briefly, the interaction term plus the main effects for depth ($\widehat{\beta }$ = − 1.60; 95% CI − 1.88 to − 1.33; Table 2) and fetch ($\widehat{\beta }$ = 0.243; 95% CI − 0.266 to 0.766; Table 2; Fig. 2a) suggest that *N. obtusa* occupancy would be expected to (1) decrease with increasing depth but most so when fetch is relatively high and to (2) increase with increasing fetch at shallower depths but decrease with increasing fetch at deeper depths.

*Nitellopsis obtusa* was significantly more likely to occupy locations closer to accesses ($\widehat{\beta }$ = − 1.12; 95% CI − 1.66 to − 0.601 for *distance to the nearest access*; Table 2; Fig. 2b). However, we did not find sufficient evidence to conclude that the *numbers of public accesses within 1 km* ($\widehat{\beta }$= − 0.208; 95% CI − 0.680 to 0.253; Table 2) or of *private accesses within 1 km* ($\widehat{\beta }$= − 0.0333; 95% CI − 0.356 to 0.291; Table 2) were consistently associated with occupancy. *Nearest access type* was nearly significantly positive ($\widehat{\beta }$ = 0.871; 95% CI − 0.0892 to 1.82; Table 2), with higher log-odds of occupancy associated with a private nearest access.

In Deviation 1, in which five lakes with no *N. obtusa* detections in our data set (Table 1) were removed from the base model, no coefficients changed by much except for the overall intercept within **β**, which increased to − 4.81 (95% CI − 6.37 to − 2.98; Table 2). This corresponds logically to a higher predicted occupancy when lakes with very low apparent occupancy are removed and also suggests these lakes did not exert leverage over the base model’s coefficients. In Deviation 2, the coefficient for *nearest access type* decreased to 0.425 (95% CI − 0.537 to 1.39; Table 2), indicating that this predictor’s near-significance in our base model was perhaps largely a consequence of Wind Lake’s inclusion (i.e., the lake with the most infested area near a private access in our data set). In Deviation 3, in which an interaction term between *distance to the nearest access* and *time since first infestation* was added to the base model, this new term was significant ($\widehat{\beta }$= 0.681; 95% CI 0.0723 to 1.30; Table 2), indicating a weakening of the negative relationship between *distance to the nearest access* and *N. obtusa* occupancy over time. The main effect for *distance to the nearest access* also shifted lower, decreasing to − 1.50 (95% CI -2.17 to − 0.843; Table 2), indicating that the negative relationship between occupancy and this predictor may be steeper immediately after an infestation begins than our base model indicates, though it then may erode with time. These results suggest that the main effect coefficient in our base model for this predictor reflects an average effect across the sampled time points post-invasion.

**Figure 3:**
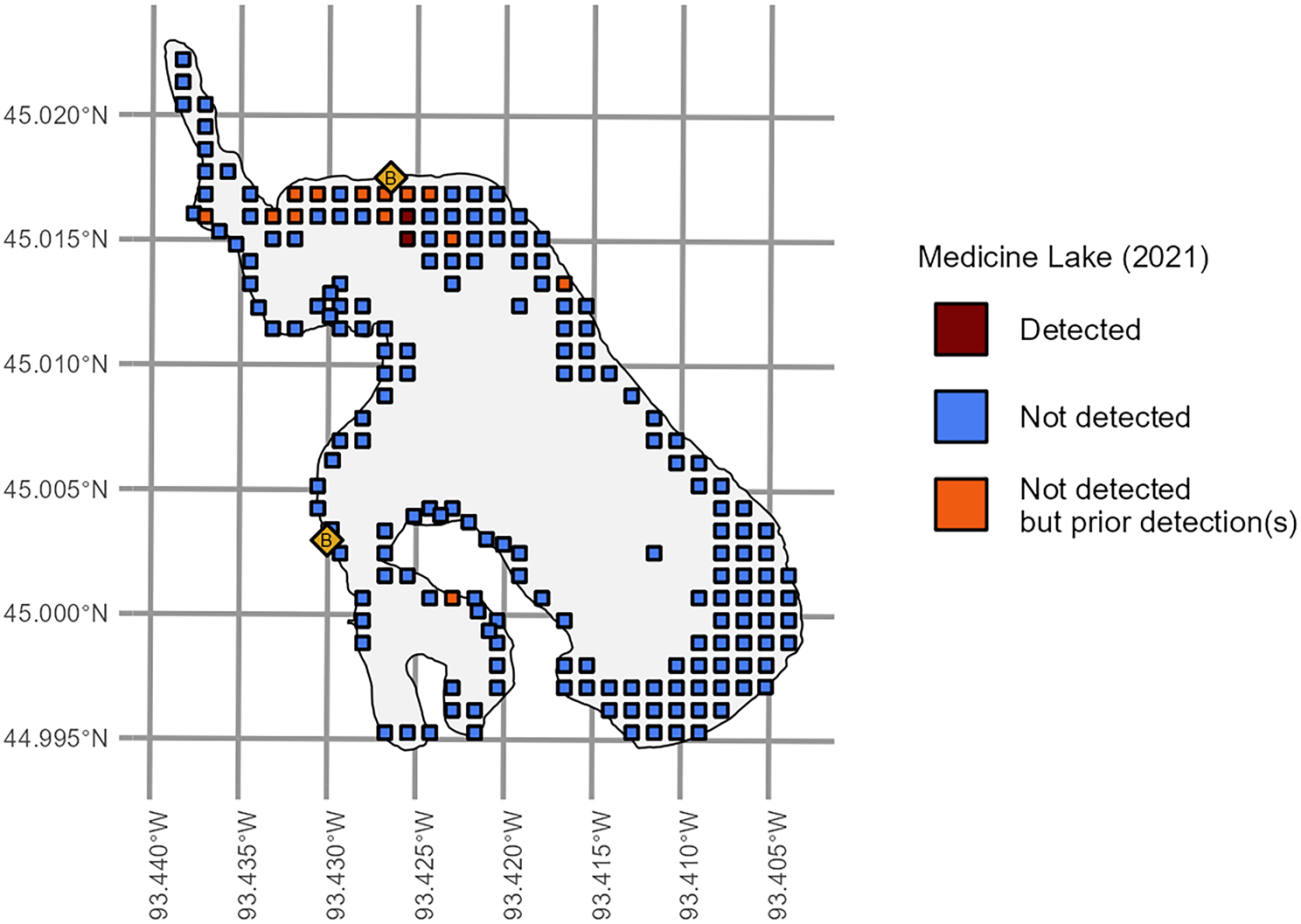
Starry stonewort (*Nitellopsis obtusa*) detection/non-detection data from point-intercept aquatic plant surveys from Medicine Lake in Minnesota, USA, between 2018 and 2021, relative to 2021 data. Orange boxes indicate where starry stonewort was not detected in 2021 but was detected in one or more previous survey event(s) at the same locations. Yellow diamonds indicate public boat accesses.

The probability of *N. obtusa* detection was negatively associated with overall *plant density* ($\widehat{\alpha }$ = − 0.348; 95% CI − 0.444 to − 0.253; Table 2) and *Characeae density* ($\widehat{\alpha }$ = − 0.165; 95% CI − 0.239 to − 0.0910; Table 2). Also as expected, the coefficient for *day of the year* was positive ($\widehat{\alpha }$ = 0.0340; 95% CI − 0.0368 to 0.107; Table 2) but was not statistically significant. The posterior mean for the overall intercept term within **α** was 0.534 (95% CI 0.419 to 0.651; Fig. 3). With *day of year*, *plant density*, and *Characeae density* set to scaled means of 0, this value would correspond to an average detection probability of ~63%, which would then decrease as either density-related predictor increased above its mean. Curiously, all three main-effect coefficients within **α** shifted in Deviation 2 (Table 2), indicating that the observation process may have been somehow unusual at Wind Lake compared to at all other lakes and that *N. obtusa* detection might actually be expected to decline later in the year at most lakes.

**Figure 4:**
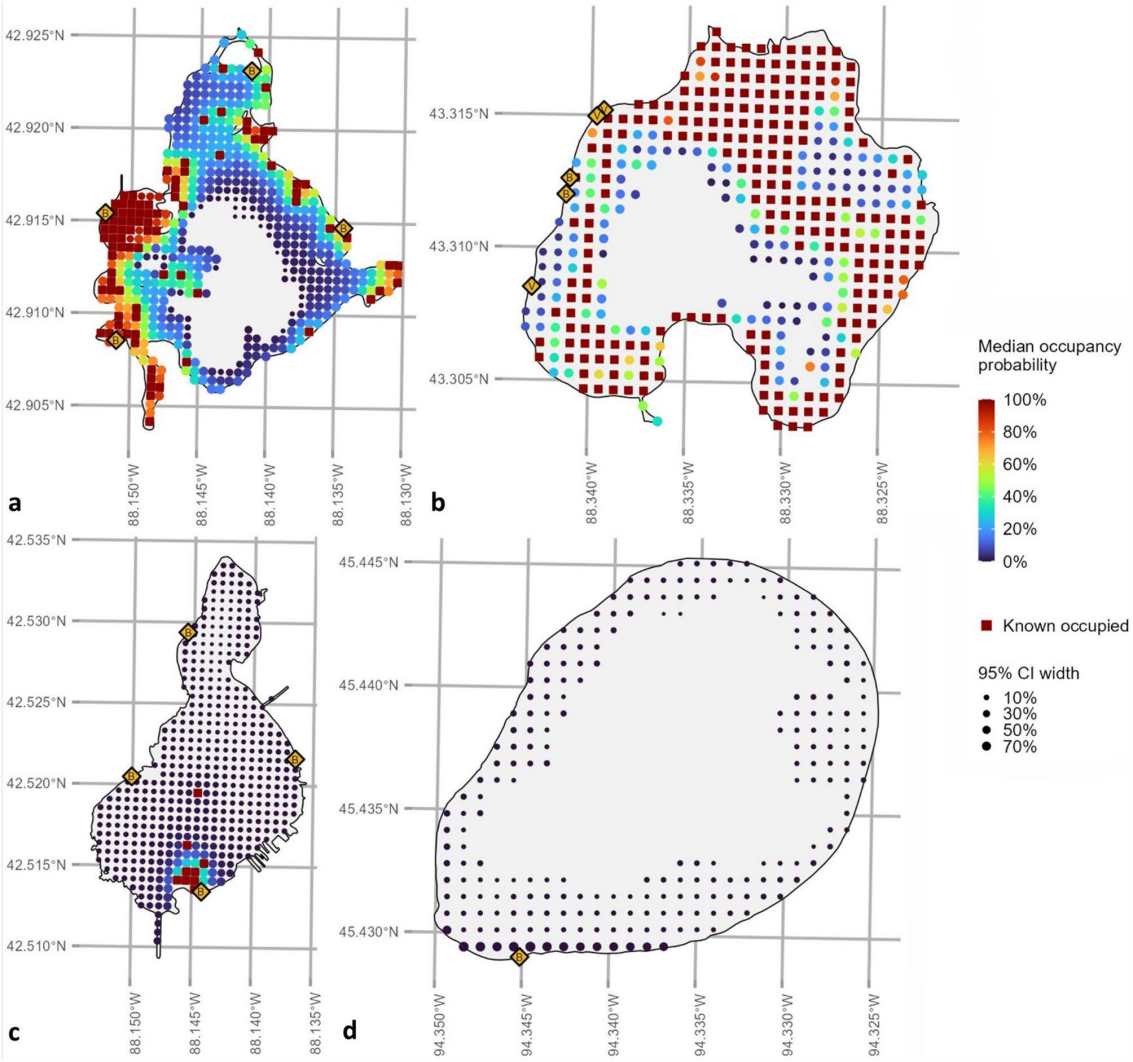
Predicted median occupancy probabilities for starry stonewort (*Nitellopsis obtusa*) for (**a**) Little Muskego Lake (Wisconsin, USA), (**b**) Pike Lake (Wisconsin, USA), (**c**) Camp Lake (Wisconsin, USA), and (**d**) Grand Lake (Minnesota, USA) from a multi-season, single-species occupancy model. These lakes typify the four qualitative classes of lakes noted in our analyses: moderate to high average occupancy probability but high uncertainty; moderate to high occupancy probability but moderate uncertainty; low to moderate occupancy probability and modest uncertainty; and low occupancy probability and low uncertainty, respectively. Point size is a function of the uncertainty around the median predicted occupancy probability at that location, as measured by the width of the 95% Credible Interval. As such, large points reflect greater uncertainty. Dark red squares indicate sites at which starry stonewort has been detected at least once. Yellow diamonds indicate boat accesses (B = Public; V = Private).

From our model results, we grouped lakes into one of four qualitative classes based on two characteristics: their predicted 2021 occupancy probability averaged across all points, and the width of the 95% CI around that predicted average occupancy probability (upper bound minus lower bound). These four classes were: (1) Three lakes with moderate-to-high predicted mean occupancy probabilities (i.e., mean $\Psi$ s > 0.349) but relatively large uncertainty around those predictions (e.g., Little Muskego Lake [mean $\Psi$: 0.368, 95% CI 0.304 to 0.436; Fig. 4a)]); (2) Six lakes with moderate-to-high predicted mean occupancy probabilities (mean $\Psi$ s between 0.160 and 0.907) but moderate uncertainty (e.g., Pike Lake [mean $\Psi$: 0.587, 95% CI 0.540 to 0.643; Fig. 4b)]); (3) Nine lakes with low-to-moderate mean predicted occupancy probabilities (mean $\Psi$ s between 0.006 and 0.115) and modest uncertainty (e.g., Camp Lake [mean $\Psi$: 0.028, 95% CI 0.015 to 0.046; Fig. 4c)]); and (4) Five lakes with both low predicted mean occupancy probabilities (i.e., mean $\Psi$ s < 0.010) and low uncertainty (e.g., Grand Lake [mean $\Psi$: 0.003, 95% CI 0.001 to 0.012; Fig. 4d)]).

## Discussion

Our within-lake occupancy model for *N. obtusa* yielded several notable results: (1) *N. obtusa* detectability was moderate and a function of the density of other macrophyte taxa present, especially other Characeae; (2) dispersal processes (especially proximity to boat accesses) were associated with *N. obtusa* occupancy; and (3) depth and fetch related to occupancy, although fetch’s relationship with occupancy was complex rather than negative as had been observed in previous studies.

Consistent with previous research^12,13,16,26^, we found a positive association between *N. obtusa* occupancy and proximity to boat accesses. These results align with *N. obtusa* not being in equilibrium in our region and boat-mediated dispersal dictating its occupancy pattern to a large extent^6^. Distance from a boat launch was also one of several variables differentiating *N. obtusa*-occupied sites from unoccupied sites among 60 lakes in Ontario, Canada^26^. Also, both distance to the nearest marina and dock density predicted *N. obtusa* occurrence within a coastal wetland of Lake Ontario^16^. As such, our results are consistent with past observations and support the notion that early detection and monitoring should preferentially occur close to accesses. More generally, our results highlight the importance of including dispersal processes in occupancy models for invasive species^10^.

Two questions remain with respect to the association between *N. obtusa* occupancy and accesses, however. First, does this association indicate *only* that *N. obtusa* depends on boat dispersal to colonize new lakes and new areas within lakes (a “crime of opportunity”)? Or are sites near accesses additionally (or instead) preferentially *habitable* for *N. obtusa* (a “crime of passion”)? It is plausible that areas around accesses might be preferentially habitable for *N. obtusa*. Accesses are often located within embayments where wind-swept *N. obtusa* fragments could aggregate and establish. Macrophytes are also regularly fragmented or disrupted by boat motors near access points, which would promote ruderal species like *N. obtusa* with high disturbance tolerance. Due to human actions, substrates may also be altered around accesses (e.g., for swimming), which could somehow favor *N. obtusa*^13,16^.

Conversely, if areas around accesses are not particularly hospitable for *N. obtusa*, we would expect a significant interaction between *time since first infestation* and *distance to the nearest access*. When we added such an interaction term to our model (Deviation 3), the term was significantly positive ($\widehat{\beta }$ = 0.681; 95% CI 0.0723 to 1.30; Table 2), indicating a weakening over time of the negative association between occupancy and *distance to the nearest access*. This suggests that *N. obtusa* spreads beyond accesses into presumably more habitable zones over time, which is more consistent with the “crime of opportunity” hypothesis. That said, determining whether habitats around accesses are key for successful *N. obtusa introduction*, successful *establishment*, or both has monitoring and management implications and should be prioritized in future work.

Second, why does occupancy vary so greatly near different accesses? Clearly, accesses are not equally “risky,” and access type (“public” versus “private”) as defined here is a crude (though perhaps not unuseful) differentiator of accesses in terms of riskiness. A multitude of factors—*e.g.*, proximity by car to other infested lakes, boater usage rates and behaviors, spread prevention programs, etc*.*—could lead to differential risk amongst accesses. While some of these factors might vary systematically between public and private accesses, we lack data to support this notion. One particular private access type (marinas) has been previously shown to positively associate with *N. obtusa* occupancy^16^, but we did not discriminate between types of private accesses because of insufficient replication. Relating occupancy with specific access characteristics, perhaps for a more common aquatic invader, could help elucidate variation in risk among accesses. Still, our results suggest that there may be opportunities to reduce introduction risk by extending spread prevention efforts commonly employed at public accesses to private ones—which appear to be *at least as* risky as public accesses are.

Our model indicated *N. obtusa* occupancy was lower at greater water depths, corroborating results from past studies^1,2,13,14,16^. However, our results indicated a more complex relationship between occupancy and fetch, one dependent on depth and positive at low depths, which contradicts some past observations. For example, while mean depths were comparable between *N. obtusa*-occupied and unoccupied sites in a large coastal wetland, mean fetches were distinct, with occupied sites having lower fetches^16^. Shallow waters correspond to higher light availability and low fetches correspond to lower-energy, less wind-mixed waters^16^. Most Characeae are thought to favor both characteristics, so it is curious we did not observe a negative association between occupancy and fetch (except at higher depths). Given our limited number of lakes and that *N. obtusa* is not at equilibrium in our region^6^, we remain uncertain regarding the true relationships between *N. obtusa* occupancy, depth, and fetch. In particular, we do not know whether these associations reflect true habitat preferences, colonization dynamics, or both.

With respect to biotic and movement-related processes, we recommend that efforts to detect *N. obtusa* preferentially target areas of shallow depth, of high fetch, and that are near accesses as these areas seem likeliest to yield detections^1^. Near-shore/near-access areas are already targeted for aquatic invasive species monitoring^27^, and our study provides support for this practice. We also note that our model’s occupancy predictions could be used to usefully classify lakes, like we have done here (Fig. 4), for different management and monitoring objectives. For example, lakes with high predicted occupancy and low uncertainty may not require as much monitoring but could be targeted for control and spread prevention, whereas lakes with low predicted occupancy but high uncertainty could be targeted for more intensive monitoring.

Our model results support the view that *N. obtusa* can be difficult to detect, even for professionals sampling known-infested lakes^16^, with an approximately 2 in 3 chance of detection with a single rake toss in an occupied location. Further, our model detected a negative association between detection probability and the density of other Characeae, suggesting *N. obtusa* is more difficult to detect when it co-occurs with other Characeae (although the mean effect size of -0.165 is relatively small on the logit scale, corresponding to a max change in probability of ~ 4.1% per standard-deviation change in Characeae density). This may be due to small amounts of *N. obtusa* being missed among larger clumps of superficially similar Characeae—especially when its highly diagnostic reproductive structures (star-shaped bulbils) are absent.

We also detected a negative association between detection probability and overall plant density (with a mean effect size of -0.348 on the logit scale corresponding to a max change in probability of ~ 8.7% per standard-deviation change in plant density). The physiological needs of macrophytes are broadly similar; locations habitable for one taxon may frequently be habitable for many^13^, and native and invasive abundances tend to positively covary^1^. Given the likelihood of *N. obtusa* density being limited by resource competition and that space on rake heads for macrophyte biomass is limited, *N. obtusa* is likely difficult to *capture* when it is co-occurring with abundant interspecific vegetation^12^, let alone then *detect* in such circumstances. Sample *timing* may also impact detection. Bulbils are only reliably produced by established beds^14^, making nascent populations harder to discover. Additionally, *N. obtusa* increases in abundance from mid-summer through late fall^28^, and our model predicted an increase in detection probability with increasing *day of year* from June through September, although this trend was not statistically significant.

Brainard and Schulz^12^ raised two more explanations for imperfect detection of *N. obtusa*: (1) *N. obtusa* could actually prefer deeper waters, where it could then be harder to recover via rake sampling methodologies^29^ and (2) because bulbils largely form in the sediment, a rake could fail to capture them if good substrate contact is not achieved. In line with their first hypothesis, we found a negative association between occupancy and depth. Without including depth in the model in both the occupancy and detection levels (which would present significant convergence challenges), our model had limited power to discriminate between lower occupancy versus lower detection with increasing depth^29–32^. *Nitellopsis obtusa* is capable of living in relatively deep waters in its native range^11^ and has even been found to prefer greater depths in one North American study^26^. Resolving *N. obtusa*’s occurrence and detectability patterns with respect to depth should be a priority in future work.

We acknowledge our model does not include all factors that may influence detection of *N. obtusa*. Selecting, quantifying, and modeling appropriate detection predictors can be challenging^29^. For example, predictors such as abundance and substrate were used in previous studies^29,32^ but were not included in our model because of a lack of suitable data or a modeling framework to incorporate them. Measures of water clarity (e.g., Secchi depth) would also likely be valuable but were unavailable at the within-lake level and likely too variable to be durable anyhow. Accounting for abundance’s near-certain impact on detection via proxy variables is likely necessary in the future but was not feasible using our data set^32^. A study aimed *exclusively* at explaining variance in *N. obtusa* detection would be a valuable next step.

We offer the following recommendations to surveyors to increase *N. obtusa* detection probability, especially early in the invasion process. First, *N. obtusa* monitoring should be concentrated in late summer (in our region, August and September), when its biomass and bulbil abundance are highest^12,28^ and other aquatic plants may be nearing senescence. Second, especially in deep waters, surveyors should ensure the rake fully contacts the substrate and is retrieved slowly through the water column to prevent release of bulbils or small fragments. Third, especially when other taxa are abundant, duplicate or even triplicate samples may be justified (despite the time and effort required) at locations of high concern or predicted occupancy, and retaining vouchers for more thorough analysis out of the field could be justifiable. Lastly, until the major factors affecting *N. obtusa* detection are elucidated, we should assume *N. obtusa* occupation is more expansive than currently known and that depth, substrate, and other factors could influence detection in ways not currently understood.

Critically, our model’s predictions generally mirrored patterns of known occurrences. The model’s predicted occupancy probabilities visually aligned with areas of known occupancy (Fig. 4), and when we correlated predicted occupancy probability with known occupancy rates in the same lakes based on our detection data, those metrics aligned extremely well (Spearman’s *ρ* = 0.848). Similar to Tucker et al.^1^, our aim was not to create the “most accurate possible” model per se but rather the most *actionable* one^2^. Our model used only a few consistently available and accessible predictors, several of which could even be crudely assessed visually during a monitoring survey but none of which would require measurement “on the spot” to be useful. Our suite of predictors also encompassed all three components of the **BAM** framework^33^, including **B**iotic factors (*e.g.*, *plant density*), **A**biotic factors (*e.g.*, *fetch*), and **M**ovement factors (*e.g.*, local access density). We accounted for (and partially explained) imperfect detection by including repeated samples and detection probability covariates and avoided errant conclusions by using systematically collected (rather than opportunistic) data^9^. Hence, we are confident that use of our model could bolster detection of new *N. obtusa* infestations.

## Materials and methods

### Study system and data

Our data come from systematic, point-intercept (PI) littoral aquatic plant surveys conducted using consistent rake sampling methodologies by the Minnesota and Wisconsin Departments of Natural Resources, parks districts, and private consultants from 2014 to 2021. For detailed protocols, see Mikulyuk et al.^19^ for Wisconsin and Perleberg et al.^34^ for Minnesota.

Briefly, sampling locations (hereafter, “points”) are established within each lake by overlaying the lake polygon with a grid. For Minnesota, sampling points are restricted to depths ≤ 15 ft. (4.57 m), and the number of points per lake is based solely on lake area, with ideally 65 m between points (i.e., 1 point/acre^34^). For Wisconsin, grids cover the entire lake, with spacing varying by lake size, littoral area, and shoreline complexity^19^. Additionally, in Wisconsin, the extent of the littoral zone is determined dynamically during a survey by finding the maximum depth of observed plant growth, with points at greater depths then excluded. For both states, points too shallow for boat navigation during a survey are not sampled.

We compiled all available surveys from all known *N. obtusa*-infested lakes in our region, yielding 75 surveys of 23 waterbodies: 7 in Minnesota and 16 in Wisconsin, USA (Fig. 1; Table 1). Most lakes were surveyed across multiple years, and a few were surveyed multiple times within some years, enabling accounting for imperfect detection (Table 1). Briefly, during a survey, a two-sided metal rake head attached to a rope or pole was lowered at each point, dragged, and retrieved. Ordinal abundance values were recorded for every taxon recovered (0 = absent, 1 =  < 25% tine coverage, 2 = 25–75% coverage, and 3 =  > 75% coverage). We converted these data to their midpoints for analysis (proportions of 0, 0.125, 0.5, and 0.875, respectively). Water depth was also recorded at each point. We restricted our analysis to points < 9.14 m (< 30 ft.) deep because (1) *N. obtusa* has been consistently recorded at relatively shallow depths in North America (≤ 7 m^15,16,35^), (2) there were no *N. obtusa* observations > 8.54 m in our dataset, and (3) the two states differ in whether they sample deeper waters. We retained 22,795 point-level sampling events after this adjustment (98% of the original point-level data).

### Predictors

We compiled locations for all public accesses for motorized boats on our study lakes or directly connected waterbodies using state databases maintained for Minnesota (https://gisdata.mn.gov/dataset/loc-water-access-sites; downloaded May 2021) and Wisconsin (https://data-wi-dnr.opendata.arcgis.com/datasets/wi-dnr::public-boat-access-sites-1/about; downloaded January 2022). We then used Google Earth Pro (version 7.3.4; Google LLC, Mountain View, CA) to locate multi-user private accesses based on location icons and names (*e.g.*, “resort”, “boat club”, “yacht club”, etc*.*) and inspected aerial imagery to confirm all accesses were intended for motorized boats and present prior to *N. obtusa* being detected in the lake. We geotagged accesses to the nearest point on the (connected) lake’s polygon. Using the R packages *tidyverse*, *sf*, and *terra*^36–39^, we derived four access-related predictors for every point: (1) *Distance* (“as the boat travels,” i.e., along the shortest path not crossing land) *to the nearest access* (of either type); (2) *Nearest access type* (private = 1, public = 0); and (3) & (4) *Numbers of public and private accesses within 1 km*, also measured “as the boat travels” ^16^.

We also derived maximum *fetch* values for every sampled point. Lake-level fetch can be defined as the maximum distance, across all bearings, that wind could travel from shoreline to shoreline unimpeded by land, reflecting maximum potential wave action. From this, we defined a fetch value for each point by forcing lines across 36 equally spaced bearings (in 10° intervals) through each point and choosing the distance of the longest such line using the *sf* package^36^.

Overall *plant density* and *Characeae density* were calculated for every observation by summing the rake coverage for all observed non-*N. obtusa* taxa and non-*N. obtusa* Characeae (*Chara*, *Nitella*, and *Tolypella* spp.), respectively. Other predictors included *time (years) since first infestation* of a lake (Table 1) and *day of the year*. All quantitative variables were mean-centered and scaled prior to analysis.

### Data analysis

To test our hypotheses, we constructed a multi-season, single-species occupancy model in a Bayesian framework using the *spOccupancy* package^40^ in R. To balance simplicity and realism, we made the following assumptions: (1) Due to sampling vagaries, variation in local *N. obtusa* density, varying experience of surveyors, etc*.*, sampling events in occupied locations can yield non-detections; (2) Because the surveyors were professionals trained to distinguish *N. obtusa* from its relatives when either were found, the number of erroneous positive detections of *N. obtusa* in our data set is negligible; and (3) There may be residual spatial autocorrelation in occupancy unaccounted for by our predictors, so spatial dependency would need to be modeled explicitly at both fine (i.e., within-lake) and broad (i.e., across lakes) scales to account for this.

Let z_*ijt*_ be the true occupancy state of sampling point *i* in lake *j* during year *t*, i.e., z_*ijt*_ equals 1 if *N. obtusa* is present at point *i* in lake *j* during year *t* and 0 otherwise. We modeled each z_*ijt*_ as a Bernoulli-distributed random variable:

1 $$z_{ijt} \sim {\text{ Bernoulli}}\left( {\Psi_{ijt} } \right)$$

where *Ψ*_*ijt*_ is the probability point *i* is occupied during year *t* and is modeled as a logit-linear function of predictors:

2 $${\text{logit}}\left( {\Psi_ {\text{ijt}}} \right) = x_{ijt} \beta + w_{ij} + \gamma_{j}$$

where $\gamma_j \sim N(0, \sigma^2_{lake}),$ **x**_*jjt*_ is a matrix containing the overall intercept as well as covariate values at point *i* and in lake *j* during year *t* for *depth*, *distance to the nearest access*, *nearest access type*, *public* and *private accesses within 1 km*, *fetch*, a *fetch by depth* interaction (to allow for non-monotonic habitat suitability relationships^6^), and *years since first infestation*; ***β*** is a matrix of fixed-effect coefficients (including an overall intercept), *w*_*ij*_ is the value of a spatial random effect at point *i* that accounts for fine-scale spatial autocorrelation, and ${\gamma }_{j}$ is an unstructured random effect of lake to account for broad-scale spatial variability in occupancy across lakes. We constructed all occupancy covariates other than year by taking their median at each point across all time periods; although depth fluctuated between separate surveys at the same point, we attributed this variation largely to spatial measurement error.

Given the large number of points in our data set (8,419; Table 1), we modeled the spatial random effect *w*_*ij*_ using a Nearest Neighbor Gaussian Process (NNGP^41^), a computationally efficient approximation to a full spatial Gaussian Process using a reduced set of nearest neighbors. Here, we used 15 neighbors following Datta et al.^41^. Briefly, an NNGP yields a multivariate normal prior for spatial random effects with mean 0 and covariance matrix $\Sigma$**,** where covariance between the spatial random effects is determined by the distances between points, a spatial variance parameter ${\sigma }^{2}$, and a spatial decay parameter $\phi$, which controls the range of the spatial autocorrelation**.** See Datta et al.^41^ and Doser et al.^40^ for details.

Let *y*_*ijkt*_ equal 1 if *N. obtusa* was detected at point* i* in lake *j* in year *t* during sampling visit *k*, and let it equal 0 otherwise. We assumed *y*_*ijkt*_ was distributed similarly to true occupancy according to:

3 $$y_{ijkt} \sim {\text{ Bernoulli}}\left( {{\text{p}}_{ijkt} \times \, z_{ijt} } \right),{\text{ with}}$$

4 $${\text{logit}}\left( {{\text{p}}_{ijkt} } \right){ } = { }{\mathbf{v}}_{{{\text{ijkt}}}} {{\varvec{\upalpha}}},$$

where **v**_ijkt_ is a matrix containing the overall intercept and covariate values at point *i* in lake *j* in year *t* during visit *k* for *day of year* and *Characeae* and *plant density* and where **α** is a matrix of fixed-effect coefficients. z_*ijt*_ is included in Eq. (3) to model the assumption that detection of *N. obtusa* is only possible at truly occupied locations.

For coefficients within ***β*** and ***α***, we used Normal(mean = 0, variance = 2.72) priors, resulting in near-uniform distributions after inverse-logit transformations. For spatial decay ($\phi$) of the spatial random effects, we used an informative uniform prior of $\left( {\frac{3}{\frac{X}{2}},\frac{1}{W}} \right)$, where X equaled the maximum observed distance between any two points in the same lake (12,135 m) and W equaled the largest minimum distance between any two points in the same lake (112 m). This restricted the spatial random effects to account primarily for within-lake spatial autocorrelation, whereas the lake random effect accounted for broad-scale variation in occupancy probability across lakes. This approach was necessary to account for both resolutions of spatial autocorrelation, as a model with just the spatial random effect alone with vague priors could not distinguish between the two resolutions and as a result failed to converge. We used a weakly informative uniform distribution of (0.001, 10) for the spatial random effects variance term (σ^2^), which accounted for spatial autocorrelation while minimizing confounding with the occupancy intercept term and preventing unreasonably large estimates on the logit scale^42^. The unstructured, lake-level random effect variance ($\sigma^2_{lake}$) was not confounded with the intercept, and so we specified a vague inverse-Gamma prior with shape and scale parameters equal to 0.1 for this term.

We fit the model using Markov chain Monte Carlo (MCMC) in the *spOccupancy* R package^40^. We ran three chains each with 250,000 samples with a burn-in period of 25,000 samples. These were then thinned to retain only 1 out of every 50 samples, resulting in 13,500 posterior samples. We assessed convergence using visual assessment of MCMC chains and Gelman-Rubin scale reduction factors (Rhats), which we required to be ≤ 1.02 for all nonspatial parameters and < 1.05 for all spatial random-effect parameters. We report posterior means as point estimates for parameters and used the (0.025, 0.975) quantiles of the posterior distribution to form 95% Credible Intervals (CIs) for these estimates. Parameters for which 0 falls outside the 95% CIs were considered statistically significant.

Post hoc, we ran three deviations from the model described above to assess our model’s sensitivity to the parameters and data included and to supplement interpretation. In the first (“Deviation 1”), we removed data from five lakes lacking any *N. obtusa* detections (Table 1) to assess whether they had undue influence on parameter estimates. In our second deviation, we removed data from Wind Lake in Wisconsin, USA (“Deviation 2”), a lake with a large *N. obtusa* infestation near a private access and much less occurance near a public access, to assess that lake’s influence on access-related parameters. Lastly, in “Deviation 3,” we added an interaction between *time since first infestation* and *distance to the nearest access* to the occupancy level of our model, as described in Eq. (2), to test whether the relationship between distance to the nearest access and occupancy weakened over time.

## Data Availability

The code and data described in this manuscript are available here: https://hdl.handle.net/11299/250199.
